# MUC4基因突变在经典型阵发性睡眠性血红蛋白尿症患者血栓事件中的作用及临床意义

**DOI:** 10.3760/cma.j.issn.0253-2727.2023.07.007

**Published:** 2023-07

**Authors:** 颖莹 陈, 惠 刘, 丽燕 李, 丽娟 李, 化泉 王, 嘉 宋, 玉红 吴, 晶 关, 莉民 邢, 国锦 王, 文 瞿, 鸿 刘, 晓明 王, 宗鸿 邵, 蓉 付

**Affiliations:** 天津医科大学总医院，天津 300052 Tianjin Medical University General Hospital, Tianjin 300052, China

**Keywords:** 阵发性睡眠性血红蛋白尿症, 基因，MUC4, 血栓事件, Paroxysmal nocturnal hemoglobinuria, Gene, MUC4, Thrombotic events

## Abstract

**目的:**

探讨MUC4基因突变在经典型阵发性睡眠性血红蛋白尿症（PNH）患者血栓事件中的作用及临床意义。

**方法:**

回顾性分析2018年6月至2022年2月天津医科大学总医院血液科收治的45例经典型PNH患者的临床资料和基因测序结果，总结经典型PNH患者MUC4基因突变情况，分析经典型PNH患者血栓事件的危险因素，明确MUC4基因突变对经典型PNH患者血栓事件累积发生率和生存的影响。

**结果:**

经典型PNH合并、不合并血栓事件患者的MUC4突变检出率分别为68.8％（11/16）、10.3％（3/29）（*P*<0.001），突变均发生在2号外显子。MUC4突变（*OR*＝20.815，*P*＝0.010）是经典型PNH患者血栓事件的独立危险因素，MUC4突变组和无MUC4突变组血栓事件累积发生率分别为78.6％（11/14）和16.1％（5/31），两组比较差异有统计学意义（*P*<0.001）。生存分析显示合并、不合并血栓事件患者总生存（OS）率分别为（34.4±25.2）％、（62.7±19.3）％（*P*＝0.045）；合并、不合并MUC4突变患者的OS率分别为（41.7±29.9）％、（59.1±18.3）％（*P*＝0.487）。

**结论:**

MUC4基因突变的经典型PNH患者血栓事件发生率高，MUC4突变是经典型PNH患者血栓事件的独立危险因素。MUC4基因突变可作为新的评估经典型PNH患者血栓形成风险的预测因素。

## Abstract (en)

**Objective:**

This study aimed to investigate the role and clinical significance of MUC4 gene mutations in thrombotic events in patients with classic paroxysmal nocturnal hemoglobinuria（PNH）patients.

**Methods:**

A retrospective analysis was conducted on the clinical data and gene sequencing results of 45 patients with classic PNH admitted to the Department of Hematology, Tianjin Medical University General Hospital, from June 2018 to February 2022. MUC4 gene mutations in patients with classic PNH were summarized, and the risk factors for thrombotic events in these patients were analyzed. Additionally, the effects of MUC4 gene mutations on the cumulative incidence and survival of thrombotic events in patients with classic PNH were determined.

**Results:**

The detection rate of MUC4 gene mutations in patients with classic PNH who experienced thrombotic events（thrombotic group）was 68.8％（11/16）, which was significantly higher than that in the non-thrombotic group［10.3％（3/29）］（*P*<0.001）. All mutations occurred in exon 2. MUC4 mutation（*OR*＝20.815, *P*＝0.010）was identified as an independent risk factor for thrombotic events in patients with classic PNH. The cumulative incidence of thrombotic events was 78.6％（11/14）in the MUC4 gene mutation group（mutation group）and 16.1％（5/31）in the non-mutation group, showing a statistically significant difference between the two groups（*P*<0.001）. Survival analysis showed a lower overall survival（OS）rate in the thrombotic group compared with that in the non-thrombotic group［（34.4±25.2）％ *vs*.（62.7±19.3）％］（*P*＝0.045）. The OS rate of patients was（41.7±29.9）％ in the mutation group and（59.1±18.3）％ in the non-mutation group（*P*＝0.487）.

**Conclusion:**

MUC4 gene mutations are associated with an increased incidence of thrombotic events in classic PNH patients, highlighting their role as independent risk factors for thrombosis in this population. These mutations can be considered a novel predictive factor that aids in evaluating the risk of thrombosis in patients with classic PNH.

阵发性睡眠性血红蛋白尿症（PNH）是一种后天获得性体细胞突变所致的造血干细胞膜缺陷引起的疾病，该病的三大临床特征为溶血性贫血、血栓形成和骨髓衰竭[1]。血栓形成是PNH患者死亡的主要原因[2]–[3]。目前认为抗凝治疗对PNH患者血栓形成具有一定的预防作用[4]，但即使接受标准抗凝治疗，PNH患者仍有血栓形成的风险[5]，且抗凝治疗可能导致严重出血，因此PNH患者血栓的一级预防尚存在争议[6]。

二次基因突变在PNH克隆增殖中发挥重要作用[7]，但其与PNH患者血栓事件的关系尚不明确。我中心既往研究发现PNH合并血栓患者MUC4基因突变发生率高[8]，为进一步明确MUC4基因突变在经典型PNH患者血栓事件中的作用，本研究我们回顾性分析45例经典型PNH患者临床资料，初步探索MUC4基因突变在经典型PNH患者的血栓预防策略中可能的临床价值。

## 病例与方法

1. 病例：本研究为回顾性研究，收集了2018年6月至2022年2月在天津医科大学总医院住院的45例经典型PNH患者的临床资料，所有患者诊断均符合国际PNH工作组标准[9]和《阵发性睡眠性血红蛋白尿症诊断与治疗中国专家共识》[10]。纳入标准：①经典型PNH；②于我院初次就诊；③具有完整的临床及实验室资料、全外显子测序/基因芯片测序结果和随访信息；④血栓事件诊断均有影像学证据支持。本研究关注的血栓事件仅包括确诊PNH同时或确诊后发生的血栓。

2. 基因突变检测：MUC4基因突变检测方法、数据分析方法参考文献[8],[11]，流式细胞术分选PNH患者外周血CD59^−^细胞，送检全外显子测序或靶基因目标区域测序。

3. 随访：采用门诊或电话联系的方式随访，随访起点为PNH诊断，患者出院后至少每6个月随访1次，主要随访指标包括血常规、溶血指标、PNH克隆比例和是否发生血栓事件等疾病并发症。随访截止时间为2022年11月1日，中位随访4（2，6）年。总生存（OS）定义为患者确诊PNH至死亡或随访终点的时间。

4. 统计学处理：采用SPSS 26.0及Graphpad Prism 8.2.1软件进行统计学分析。连续变量采用Shapiro-Wilk检验进行正态性分析，正态分布数据以“均数±标准差”表示，偏态分布数据以“中位数（*Q*_1_，*Q*_3_）”表示，分类变量以例数（构成比）表示。两组间连续变量组间比较采用独立样本*t*检验（正态分布）或Mann-Whitney *U*检验（偏态分布），分类变量比较采用*χ*^2^检验或Fisher确切概率法。通过受试者工作特征曲线（ROC曲线）约登指数对应的临界点作为连续性变量的截断值将连续性变量转化为二分类变量，采用Logistic回归模型进行影响PNH患者血栓事件的单因素及多因素分析，其中单因素分析*P*<0.1的指标纳入多因素模型。采用Kaplan-Meier法绘制生存曲线并计算OS率。*P*<0.05为差异有统计学意义。

## 结果

1. 临床特征：45例PNH患者中男27例、女18例，中位年龄35（20，50）岁，CD59^−^红细胞比例为（46.07±27.04）％，CD59^−^粒细胞比例为（78.21±19.80）％，CD14^−^FLAER^−^比例为（82.33±15.79）％，CD24^−^FLAER^−^比例为（84.88±16.60）％。共有16例患者发生过22次血栓事件，包括脑梗死（9次）、下肢静脉血栓（8次）、脾梗死（2次）、门静脉血栓（1次）、颅内静脉血栓（1次）和心肌梗死（1次），其中有5例下肢静脉血栓为住院期间常规筛查时发现的无症状血栓。根据患者病程中是否发生过血栓事件，将患者分为血栓组（16例，35.6％）和无血栓组（29例，64.4％）。

2. PNH患者MUC4基因突变情况：45例PNH患者中，14例伴MUC4基因突变，其中11例发生过血栓事件。共检测到11种MUC4基因突变类型，均发生在2号外显子，导致不同的氨基酸改变（表1）。血栓组患者MUC4基因突变检出率为68.8％（11/16），明显高于无血栓组的10.3％（3/29）（Fisher，*P*＝0.006）。

**表1:** 14例阵发性睡眠性血红蛋白尿症（PNH）患者检出11种MUC4基因突变类型

突变功能区	突变种类	基因改变	氨基酸改变
编码区	移码插入	c.12161_12162insAC	p.A4054fs
编码区	错义SNV	c.T8888A	p.V2963D
编码区	错义SNV	c.G7502A	p.S2501N
编码区	错义SNV	c.A7231C	p.T2411P
编码区	移码缺失	c.6928delG	p.A2310fs
编码区	非移码缺失	c.5258_5305del	p.1753_1769del
编码区	非移码缺失	c.3050_3097del	p.1017_1033del
编码区、剪接位点区	非移码插入	c.340_341insTTACGCAGGAGACAG	p.A114delinsVTQETA
编码区、剪接位点区	非移码插入	c.340_341insTTACGCAGGAGACGG	p.A114delinsVTQETA
编码区	非移码插入	c.336_337insTCTGTTACGCAGGAG	p.T113delinsSVTQET
编码区	终止突变	c.333_334insTAGACTGTTACGCAG	p.E112delinsX

**注** SNV：单核苷酸位点变异

3. PNH患者血栓事件的危险因素分析：比较了血栓组和无血栓组PNH患者的临床指标，单因素分析显示高龄、男性、高间接胆红素水平和MUC4突变是PNH患者血栓事件的危险因素（表2）。纳入单因素分析*P*<0.1的指标进行Logistic回归，结果显示年龄≥50岁、MUC4突变是PNH患者发生血栓事件的独立危险因素（*OR*＝16.924，*P*＝0.012；*OR*＝20.815，*P*＝0.010）（表3）。

**表2:** 阵发性睡眠性血红蛋白尿症（PNH）患者血栓事件的单因素分析

特征	血栓组（16例）	无血栓组（29例）	统计量	*P*值
中位年龄［岁，*M*（*Q_1_*，*Q_3_*）］	50（31, 67）	33（27, 46）	−2.623	0.006
性别［例（％）］			4.671	0.031
男	13（81.3）	14（48.3）		
女	3（18.7）	15（51.7）		
RBC（×10^12^/L，*x±s*）	2.23±0.41	2.33±0.70	−0.642	0.525
WBC［×10^9^/L，*M*（*Q_1_*，*Q_3_*）］	4.91（3.46, 7.18）	4.83（2.94, 6.35）	−0.735	0.462
HGB（g/L，*x±s*）	72.69±15.30	70.86±18.77	0.332	0.741
PLT（×10^9^/L，*x±s*）	90（28, 163）	117（60, 188）	−0.617	0.538
Ret（％，*x±s*）	8.96±4.18	7.31±3.32	1.457	0.152
LDH［U/L，*M*（*Q_1_*，*Q_3_*）］	1 397（682, 2 092）	1 233（953, 1 724）	−1.020	0.308
IBIL（µmol/L，*x±s*）	26.68±12.47	19.61±9.18	2.173	0.035
D-Dimer［µg/L，*M*（*Q_1_*，*Q_3_*）］	1 140（355, 3 189）	544（260, 1 476）	−1.683	0.092
游离血红蛋白［mg/L，*M*（*Q_1_*，*Q_3_*）］	161（29, 236）	163（89, 236）	−0.159	0.874
结合珠蛋白［g/L，*M*（*Q_1_*，*Q_3_*）］	0.09（0, 0.19）	0（0, 0.33）	−0.614	0.539
CD59^−^红细胞（％，*x±s*）	49.48±29.42	44.19±25.98	0.623	0.536
CD59^−^粒细胞（％，*x±s*）	79.23±24.14	77.66±17.41	0.252	0.802
CD14^−^FLAER^−^（％，*x±s*）	83.14±21.48	81.99±11.99	0.254	0.801
CD24^−^FLAER^−^（％，*x±s*）	84.91±22.34	84.86±12.87	0.009	0.993
MUC4基因突变［例（％）］			13.799	<0.001
有	11（68.8）	3（10.3）		
无	5（31.2）	26（89.7）		
PIG-A基因突变［例（％）］			0.048	0.826
有	11（68.7）	19（65.5）		
无	5（31.3）	10（34.5）		

**注** Ret：网织红细胞；LDH：乳酸脱氢酶；IBIL：间接胆红素；D-Dimer：D-二聚体；FLAER：嗜水气单胞菌溶素变异体

**表3:** PNH患者血栓事件的多因素分析

风险因素	*OR*（95%*CI*）	*P*值
IBIL≥27 µmol/L	1.485（0.190~11.639）	0.707
D-Dimer≥723 µg/L	0.877（0.099~7.772）	0.906
年龄≥50岁	16.924（1.864~153.679）	0.012
男性	4.330（0.401~46.802）	0.227
MUC4突变	20.815（2.080~208.278）	0.010

**注** PNH：阵发性睡眠性血红蛋白尿症；IBIL：间接胆红素；D-Dimer：D-二聚体

4. MUC4基因突变对PNH患者血栓事件累积发生率和生存的影响分析：截至随访终点，所有PNH患者血栓事件累积发生率为35.6％（16/45）。MUC4突变组14例患者中11例（78.6％）合并血栓事件，其中确诊PNH同时合并血栓3例，确诊后1年内发生血栓3例，确诊后第3年发生血栓2例，余3例分别于确诊后第8、20及29年发生血栓；无MUC4突变组31例患者中5例（16.1％）合并血栓事件，其中确诊PNH同时合并血栓患者1例，确诊后1年内发生血栓1例，确诊后第3年发生血栓2例，余1例患者于确诊后第8年发生血栓事件。MUC4突变组和无MUC4突变组血栓事件累积发生率差异有统计学意义（Fisher，*P*<0.001）。截至随访终点，45例患者中11例死亡，6例死于血栓事件（3例在第1次血栓时死亡，3例死于第2次血栓事件），2例患者死于感染，2例死于出血，1例死于心衰，死亡距离诊断的中位时间4（1，11）年。至随访终点，45例PNH患者OS率为（54.5±15.6）％，其中合并血栓事件的患者OS率为（34.4±25.2）％，无血栓组则为（62.7±19.3）％，差异有统计学意义（*χ*^2^＝4.001，*P*＝0.045）；合并MUC4突变的患者OS率为（41.7±29.9）％，无突变组为（59.1±18.3）％，差异无统计学意义（*χ*^2^＝0.484，*P*＝0.487）。

## 讨论

血栓是PNH患者常见的并发症，多为静脉血栓，腹部静脉（肝静脉、门静脉、脾静脉和肠系膜静脉）、脑部静脉和四肢静脉是栓塞最常见的部位，29％～44％的PNH患者在其疾病过程中至少发生过1次血栓事件[12]。虽然依库珠单抗可以明显降低PNH患者血栓风险[13]，但即使是接受补体抑制治疗的患者，血栓事件依然影响PNH患者生存[14]，我们的研究也证实血栓组患者OS率明显低于无血栓的患者。由于抗凝治疗疗效的不确定性及可能带来的出血风险，PNH患者血栓一级预防存在争议[6]。还有研究发现血栓的患病率在日本、中国和墨西哥低于美国和欧洲的患者，也使得来自这些种族/地理背景的PNH患者的抗凝预防很难证明是合理的[15]–[16]。因此，寻找评估血栓形成风险的可靠因素，从而识别PNH患者中血栓的高危人群，才能更好的实现基于风险分层的预防性抗血栓治疗的策略。

目前认为，较大的GPI粒细胞缺陷克隆比例、年龄在55岁以上、怀孕和产后、输血需求、急性溶血发作均与血栓形成风险增加相关[17]–[19]，本研究也证实高龄是血栓形成的危险因素。由于纳入研究的经典型PNH患者PNH克隆比例均较高，血栓组和无血栓组患者PNH克隆比例差异无统计学意义，但血栓组患者各系PNH克隆比例均高于无血栓组。我中心既往研究显示92例门诊随诊的PNH患者血栓发生率为13.58％[20]，本研究和我科另一项住院患者PNH血栓相关研究[21]中血栓发生率分别为35.6％和31.4％，这种差异可能与患者组成有关。住院治疗的PNH患者多存在急性溶血发作或严重并发症，且本研究纳入的经典型PNH患者PNH克隆比例相对较高。此外为了进一步识别血栓患者，住院期间我们会常规行血管B超等血栓相关筛查，可以发现一部分无症状血栓患者，因此住院患者统计所得血栓发生率较高。未来我们也将会进一步扩大门诊PNH患者的测序检查和血栓筛查，更准确、更全面的统计PNH患者血栓事件发生率。

PNH患者血栓形成倾向可能与多种因素有关。PIG-A基因突变造成细胞膜表面GPI锚连蛋白（GPI-AP）的缺失，补体介导的血管内溶血和血小板激活可能是血栓的始动因素，游离血红蛋白毒性、一氧化氮耗损等多种机制进一步推动了血栓的形成，纤溶系统功能缺陷、内皮细胞功能障碍和补体介导的促凝机制也参与其中[3],[12],[21]。除此之外，有研究证实二次突变基因也可能参与PNH血栓形成[8],[22]，我们发现合并血栓事件的经典型PNH患者MUC4基因突变发生率明显高于非血栓组，合并MUC4突变的患者血栓事件累积发生率也明显高于无MUC4突变患者，MUC4突变是经典型PNH患者血栓事件的独立危险因素，因此我们推测MUC4可能参与经典型PNH患者血栓形成。虽然合并MUC4突变的患者OS率低于无突变的患者，但差异无统计学意义，后续需继续扩大病例数进一步分析。MUC4基因位于3号染色体q29区域，包含26个外显子，编码一种膜结合黏蛋白，其在大鼠中的同源物为sialomucin complex（SMC）。MUC4/SMC由一个大的黏蛋白亚基（MUC4α）和跨膜亚基（MUC4β）构成[23]，掩盖在细胞表面，在空间上阻碍了细胞-细胞、细胞-细胞基质、细胞-分子的结合，发挥抗黏附作用[24]。本研究发现PNH患者MUC4突变均发生在2号外显子，其编码的串联重复序列（TR）结构域在MUC4介导的抗细胞黏附中起重要作用[25]，提示我们MUC4可能是通过影响细胞黏附参与PNH患者血栓形成。多项研究证实MUC4在多种癌症中过表达，通过其抗黏附作用触发肿瘤细胞从原位肿瘤位点分离[26]，并可以促进循环肿瘤细胞与血细胞的结合来增加循环中肿瘤细胞的存活[27]，在肿瘤转移中发挥重要作用。与癌症中MUC4过表达导致抗黏附功能增加促进肿瘤的转移相反，PNH患者MUC4突变后其表达水平下调[8]，可能导致细胞黏附功能增强，参与PNH患者血栓形成，相关机制仍需后续深入探索。

综上，本研究显示，MUC4基因突变的经典型PNH患者血栓事件发生率高，MUC4突变是经典型PNH患者血栓事件的独立危险因素。MUC4基因突变可作为新的的评估经典型PNH患者血栓形成风险的预测因素，有助于我们未来更好的实现基于风险分层的预防性抗血栓治疗策略。未来我们也将进一步在其他亚型的PNH患者和与非PNH血栓患者中确定MUC4与血栓形成的关系，并进行相关的机制研究。
